# Novel rendezvous technique for covered metal stent placement using balloon-assisted endoscope in duodenal perforation

**DOI:** 10.1055/a-2313-3265

**Published:** 2024-05-17

**Authors:** Kei Saito, Yoshitomo Ishikawa, Mai Kitahara, Shuzo Nomura, Mariko Fujisawa, Hirofumi Kogure

**Affiliations:** 1 Division of Gastroenterology and Hepatology, Nihon University School of Medicine, Department of Internal Medicine, Tokyo, Japan

Duodenal perforations pose a significant medical challenge. While endoscopic closure can be attempted 1 2 , these cases often necessitate surgical intervention 3 . In situations where surgery carries high risks, placing a covered metal stent offers a viable alternative 4 5 . This report describes the novel use of a covered metal stent, applied using the rendezvous technique, in treating a patient with duodenal perforation post-gastrojejunostomy ( Video 1 ).

Covered metal duodenal stent placement using the rendezvous technique was performed for duodenal perforation in a patient with gastrojejunostomy.Video 1

A 62-year-old woman receiving chemotherapy for ascending colon cancer experienced deteriorating symptoms of gastrointestinal obstruction caused by duodenal stenosis and ascending colon stricture. Following a gastrojejunal bypass and ileo-transverse colon bypass, she was urgently admitted for perforation in the horizontal part of the duodenum ( Fig. 1 a,b). Initial attempts at surgical repair were challenging due to the perforation resulting from direct invasion by colon cancer and the inability to locate the perforation site because of severe adhesions. Consequently, closure of the perforation was attempted using a covered metal duodenal stent. However, endoscopic stenting from the oral side was difficult because the severe stenosis prevented passage of the guidewire, and the guidewire could easily exit into the abdominal cavity from the perforation site ( Fig. 2 a). Therefore, a balloon-assisted endoscope was used to enter via the anorectal route through the gastrojejunal bypass. This approach allowed the guidewire to pass through the stenosis ( Fig. 2 b,c,d). The guidewire was then grasped from the duodenal horn side and pulled out through the forceps channel ( Fig. 3 a). A covered Nitinol duodenal stent (Taewoong Medical, Seoul, Korea) was subsequently positioned from the jejunum to the duodenal horn using the rendezvous technique ( Fig. 3 b,c). The procedure concluded after confirming the absence of leakage from the digestive tract into the abdominal cavity via contrast imaging ( Fig. 3 d). This case underscores the significance of advanced endoscopic techniques in managing patients for whom traditional surgical options are unsuitable.

**Fig. 1:**
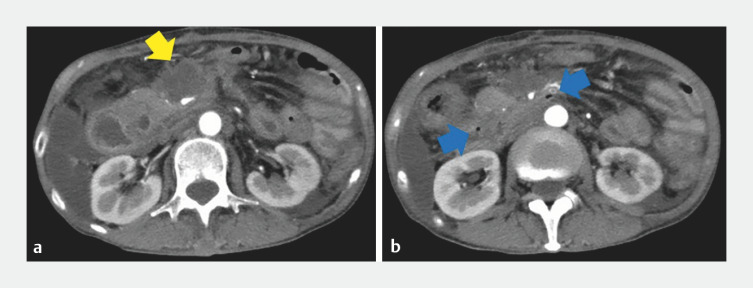
Fluid collection (yellow arrow) and free air (blue arrow) were seen around the horizontal part of the duodenum.

**Fig. 2:**
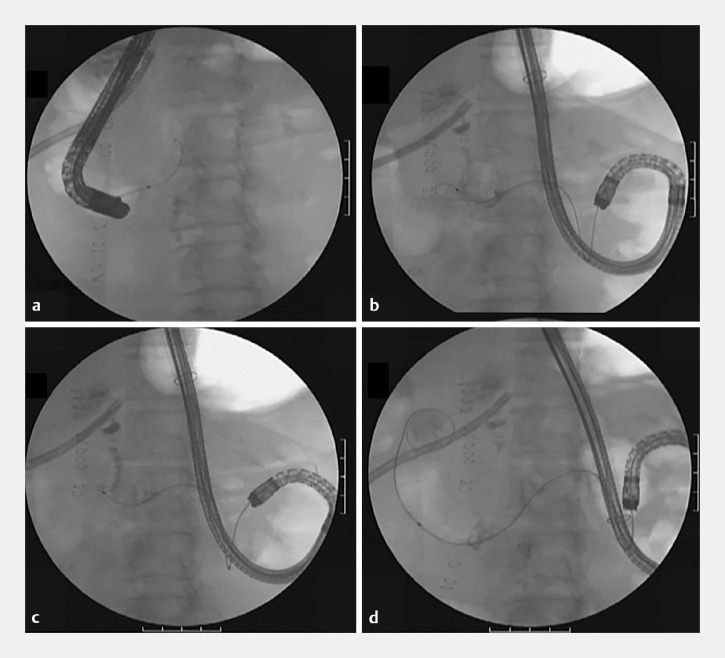
**a** The stenosis was so severe that the guidewire could not pass through. **b,c** We approached from the anorectal side through the gastrojejunal bypass. **D** The guidewire was passed through the stenosis using balloon-assisted endoscopy.

**Fig. 3:**
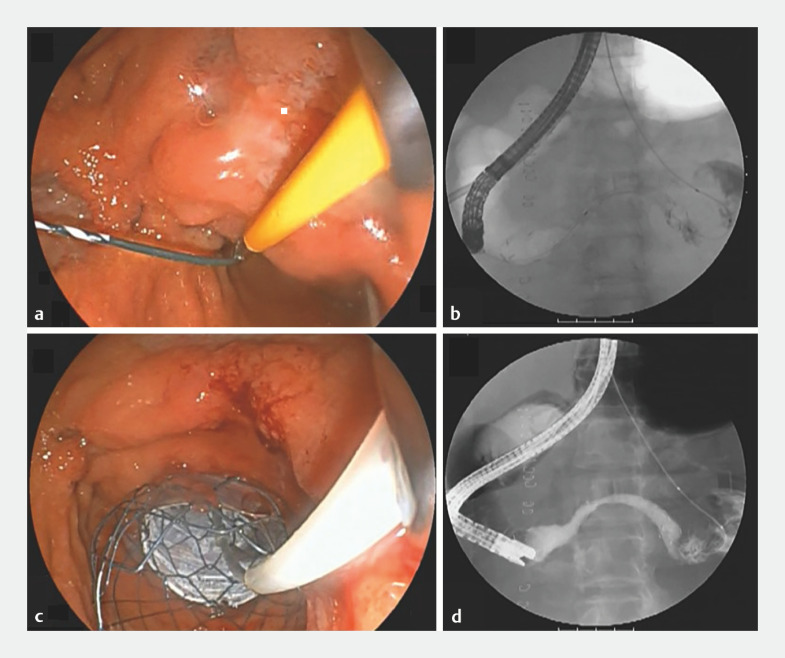
**a** The guidewire was grasped from the duodenal horn side. **b,c** A covered Nitinol duodenal stent was placed from the jejunum to the duodenal horn in a rendezvous technique. **d** No contrast medium flowed out of the gastrointestinal tract into the abdominal cavity.

Endoscopy_UCTN_Code_TTT_1AO_2AI

## Notes

Endoscopy E-Videos https://eref.thieme.de/e-videos
*E-Videos* is an open access online section of the journal *Endoscopy* , reporting on interesting cases and new techniques in gastroenterological endoscopy. All papers include a high-quality video and are published with a Creative Commons CC-BY license. Endoscopy E-Videos qualify for HINARI discounts and waivers and eligibility is automatically checked during the submission process. We grant 100% waivers to articles whose corresponding authors are based in Group A countries and 50% waivers to those who are based in Group B countries as classified by Research4Life (see: https://www.research4life.org/access/eligibility/ ). This section has its own submission website at https://mc.manuscriptcentral.com/e-videos .
